# *N*-(4-Chloro-2-methyl­phen­yl)maleamic acid

**DOI:** 10.1107/S1600536811047817

**Published:** 2011-11-16

**Authors:** K. Shakuntala, Viktor Vrábel, B. Thimme Gowda, Jozef Kožíšek

**Affiliations:** a Department of Chemistry, Mangalore University, Mangalagangotri 574 199, Mangalore, India; b Institute of Physical Chemistry and Chemical Physics, Slovak University of Technology, Radlinského 9, SK-812 37 Bratislava, Slovak Republic

## Abstract

In the mol­ecular structure of the title compound, C_11_H_10_ClNO_3_, the conformation of the N—H bond in the amide segment is *syn* to the *ortho*-methyl group in the phenyl ring. The C=O and O—H bonds of the acid group are in the relatively rare *anti* position with respect to each other. This is an obvious consequence of the hydrogen bond donated to the amide carbonyl group. The central oxobutenoic acid core C(=O)—C=C—C—OH is twisted by 31.65 (6)° out of the plane of the 4-chloro-2-methyl­phenyl ring. An intra­molecular O—H⋯O hydrogen bond occurs. In the crystal, N—H⋯O hydrogen bonds link the mol­ecules into infinite chains running along the *a* axis.

## Related literature

For studies on the effects of substituents on the structures and other aspects of *N*-(ar­yl)-amides, see: Gowda *et al.* (2000 ▶, 2010 ▶); Prasad *et al.* (2002 ▶), on *N*-(ar­yl)-methane­sulfonamides, see: Jayalakshmi & Gowda (2004 ▶), on *N*-(ar­yl)-aryl­sulfonamides, see: Shetty & Gowda (2005 ▶) and on *N*-chloro­aryl­sulfonamides, see: Gowda & Kumar (2003 ▶). For modes of inter­linking carb­oxy­lic acids by hydrogen bonds, see: Leiserowitz (1976 ▶)
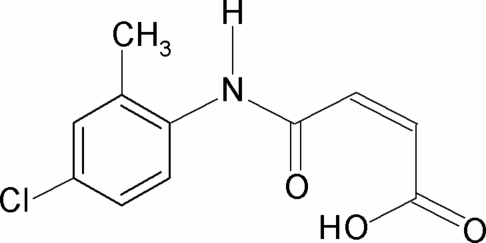


## Experimental

### 

#### Crystal data

C_11_H_10_ClNO_3_
*M* *_r_* = 239.65
Orthorhombic, 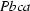

*a* = 12.1310 (11) Å
*b* = 7.3990 (7) Å
*c* = 25.466 (2) Å
*V* = 2285.7 (3) Å^3^
*Z* = 8
Mo *K*α radiation
μ = 0.33 mm^−1^
*T* = 295 K
0.45 × 0.35 × 0.25 mm

#### Data collection

Oxford Diffraction Xcalibur diffractometer
Absorption correction: multi-scan (*CrysAlis PRO*; Oxford Diffraction, 2009 ▶) *T*_min_ = 0.865, *T*_max_ = 0.918
17740 measured reflections
1819 independent reflections
1642 reflections with *I* > 2σ(*I*)
*R*_int_ = 0.025

#### Refinement

*R*[*F* ^2^ > 2σ(*F* ^2^)] = 0.033
*wR*(*F* ^2^) = 0.089
*S* = 1.02
1819 reflections
154 parameters
2 restraints
H atoms treated by a mixture of independent and constrained refinement
Δρ_max_ = 0.19 e Å^−3^
Δρ_min_ = −0.27 e Å^−3^

### 

Data collection: *CrysAlis PRO* (Oxford Diffraction, 2009 ▶); cell refinement: *CrysAlis PRO*; data reduction: *CrysAlis PRO*; program(s) used to solve structure: *SHELXS97* (Sheldrick, 2008 ▶); program(s) used to refine structure: *SHELXL97* (Sheldrick, 2008 ▶); molecular graphics: *DIAMOND* (Brandenburg, 2002 ▶); software used to prepare material for publication: *SHELXL97*, *PLATON* (Spek, 2009 ▶) and *WinGX* (Farrugia, 1999 ▶).

## Supplementary Material

Crystal structure: contains datablock(s) I, global. DOI: 10.1107/S1600536811047817/bq2317sup1.cif

Structure factors: contains datablock(s) I. DOI: 10.1107/S1600536811047817/bq2317Isup2.hkl

Supplementary material file. DOI: 10.1107/S1600536811047817/bq2317Isup3.cml

Additional supplementary materials:  crystallographic information; 3D view; checkCIF report

## Figures and Tables

**Table 1:** Hydrogen-bond geometry (Å, °)

*D*—H⋯*A*	*D*—H	H⋯*A*	*D*⋯*A*	*D*—H⋯*A*
N1—H1⋯O3^i^	0.86 (1)	2.10 (1)	2.9556 (19)	174 (2)
O2—H2*A*⋯O1	0.92 (1)	1.57 (1)	2.4797 (17)	171 (3)

Symmetry code: (i) 
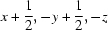
.

**Fig. 1.**
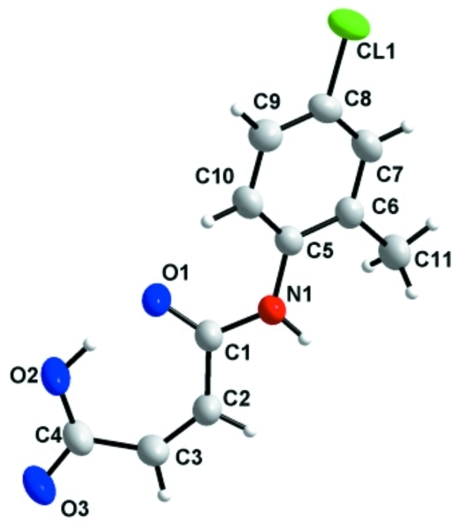
Molecular structure of the title compound showing the atom labeling scheme. Displacement ellipsoids are drawn at the 50% probability level.

**Fig. 2.**
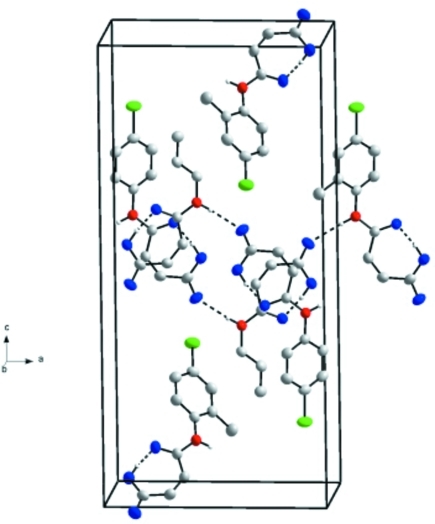
Packing view of the title compound. Molecular chains along a-axis are generated by N–H···O hydrogen bonds which are shown as dashed lines. H atoms not involved in H-bonding have been omitted.

## supplementary crystallographic information

### Comment

The amide moiety is the constituent of many biologically significant compounds. As part of our studies on the substituent effects on the structures and other aspects of *N*-(aryl)-amides (Gowda *et al.*, 2000, 2010), *N*-(aryl)-methanesulfonamides (Jayalakshmi & Gowda, 2004), *N*-(aryl)-arylsulfonamides (Shetty & Gowda, 2005) and *N*-chloroarylsulfoamides (Gowda & Kumar, 2003), in the present work, the crystal structure of *N*-(4-chloro-2-methylphenyl)-maleamic acid (I) has been determined (Fig.1).

The conformations of the N—H and the C=O bonds in the amide segment are *anti* to each other. But the conformation of the N—H bond is *syn* to the *ortho*-methyl group in the phenyl ring. In the maleamic acid moiety, the amide C=O bond is *anti* to the adjacent C—H bond, while the carboxyl C=O bond is *syn* to the adjacent C—H bond. The observed rare *anti* conformation of the C=O and O—H bonds of the acid group is similar to that observed in *N*-(2-methylphenyl)-maleamic acid (Gowda *et al.*, 2010). This is an obvious consequence of the hydrogen bond donated to the amide carbonyl group. The central oxobutenoic acid core C(=O)—C=C—C—OH is twisted by 31.65 (6)° out of the plane of the 4-chloro-2-methylphenyl ring. The C2–C3 bond length of 1.333 (2)Å clearly indicates the double bond character.

The various modes of interlinking carboxylic acids by hydrogen bonds is described elsewhere (Leiserowitz, 1976).

In (I), both the intramolecular O–H···O and intermolecular N–H···O hydrogen bonds have been observed. The packing of molecules linked by N—H···O hydrogen bonds into infinite chains running along the *a*-axis is shown in Fig. 2.

### Experimental

The solution of maleic anhydride (0.025 mol) in toluene (25 ml) was treated dropwise with the solution of 4-chloro-2-methylaniline (0.025 mol) also in toluene (20 ml) with constant stirring. The resulting mixture was stirred for about 30 min. and set aside for an additional 30 min. at room temperature for the completion of reaction. The mixture was then treated with dilute hydrochloric acid to remove the unreacted 4-chloro-2-methylaniline. The resultant solid *N*-(4-chloro-2-methylphenyl)-maleamic acid was filtered under suction and washed thoroughly with water to remove the unreacted maleic anhydride and maleic acid. It was recrystallized to constant melting point from ethanol. The purity of the compound was checked by elemental analysis and characterized by its infrared spectra.

The plate like colorless single crystals of the title compound used in X-ray diffraction studies were grown in an ethanol solution by slow evaporation (0.5 g in about 30 ml of ethanol) at room temperature.

### Refinement

All hydrogen atoms were placed in calculated positions with C–H distances of 0.93Å (C-aromatic) and 0.96Å (*C*-methyl), and constrained to ride on their parent atoms. Amide and O—H atoms were seen in difference map and were refined with the N—H and O—H distances restrained to 0.86 (1)Å and 0.92 (1) Å, respectively. The *U*_iso_(H) values were set at 1.2 *U*_eq_ (C-aromatic, N) or 1.5*U*_eq_ (*C*-methyl).

### Crystal data

**Table:**

C_11_H_10_ClNO_3_	*D*_x_ = 1.393 Mg m^−^^3^
*M**_r_* = 239.65	Mo *K*α radiation, λ = 0.71073 Å
Orthorhombic, *P**b**c**a*	Cell parameters from 1819 reflections
*a* = 12.1310 (11) Å	θ = 3.6–24.4°
*b* = 7.3990 (7) Å	µ = 0.33 mm^−^^1^
*c* = 25.466 (2) Å	*T* = 295 K
*V* = 2285.7 (3) Å^3^	Plate, colourless
*Z* = 8	0.45 × 0.35 × 0.25 mm
*F*(000) = 992	

### Data collection

**Table:**

Oxford Diffraction Xcalibur diffractometer	1819 independent reflections
Radiation source: fine-focus sealed tube	1642 reflections with *I* > 2σ(*I*)
graphite	*R*_int_ = 0.025
Detector resolution: 0 pixels mm^-1^	θ_max_ = 24.4°, θ_min_ = 3.6°
ω scans with κ offsets	*h* = −14→13
Absorption correction: multi-scan (*CrysAlis PRO*; Oxford Diffraction, 2009)	*k* = −8→8
*T*_min_ = 0.865, *T*_max_ = 0.918	*l* = −29→28
17740 measured reflections	

### Refinement

**Table:**

Refinement on *F*^2^	Primary atom site location: structure-invariant direct methods
Least-squares matrix: full	Secondary atom site location: difference Fourier map
*R*[*F*^2^ > 2σ(*F*^2^)] = 0.033	Hydrogen site location: inferred from neighbouring sites
*wR*(*F*^2^) = 0.089	H atoms treated by a mixture of independent and constrained refinement
*S* = 1.02	*w* = 1/[σ^2^(*F*_o_^2^) + (0.0445*P*)^2^ + 0.9459*P*] where *P* = (*F*_o_^2^ + 2*F*_c_^2^)/3
1819 reflections	(Δ/σ)_max_ < 0.001
154 parameters	Δρ_max_ = 0.19 e Å^−^^3^
2 restraints	Δρ_min_ = −0.27 e Å^−^^3^

### Special details

**Table:**

Geometry. All e.s.d.'s (except the e.s.d. in the dihedral angle between two l.s. planes) are estimated using the full covariance matrix. The cell e.s.d.'s are taken into account individually in the estimation of e.s.d.'s in distances, angles and torsion angles; correlations between e.s.d.'s in cell parameters are only used when they are defined by crystal symmetry. An approximate (isotropic) treatment of cell e.s.d.'s is used for estimating e.s.d.'s involving l.s. planes.
Refinement. Refinement of *F*^2^ against ALL reflections. The weighted *R*-factor *wR* and goodness of fit *S* are based on *F*^2^, conventional *R*-factors *R* are based on *F*, with *F* set to zero for negative *F*^2^. The threshold expression of *F*^2^ > σ(*F*^2^) is used only for calculating *R*-factors(gt) *etc*. and is not relevant to the choice of reflections for refinement. *R*-factors based on *F*^2^ are statistically about twice as large as those based on *F*, and *R*- factors based on ALL data will be even larger.

### Fractional atomic coordinates and isotropic or equivalent isotropic displacement parameters (Å^2^)

**Table:**

	*x*	*y*	*z*	*U*_iso_*/*U*_eq_	
C1	0.29051 (13)	0.3610 (2)	0.08033 (7)	0.0428 (4)	
C2	0.31769 (14)	0.3019 (3)	0.02624 (7)	0.0485 (5)	
H2	0.3925	0.2917	0.0188	0.058*	
C3	0.25011 (15)	0.2612 (3)	−0.01329 (6)	0.0505 (5)	
H3	0.2860	0.2317	−0.0444	0.061*	
C4	0.12845 (15)	0.2543 (3)	−0.01670 (7)	0.0496 (5)	
C5	0.37645 (12)	0.4444 (2)	0.16488 (6)	0.0368 (4)	
C6	0.46315 (13)	0.5525 (2)	0.18298 (6)	0.0383 (4)	
C7	0.46319 (14)	0.6020 (2)	0.23578 (6)	0.0431 (4)	
H7	0.5205	0.6723	0.2489	0.052*	
C8	0.37918 (14)	0.5481 (2)	0.26889 (6)	0.0410 (4)	
C9	0.29334 (14)	0.4439 (2)	0.25077 (7)	0.0449 (4)	
H9	0.2366	0.4097	0.2732	0.054*	
C10	0.29265 (14)	0.3907 (2)	0.19860 (7)	0.0429 (4)	
H10	0.2357	0.3185	0.1861	0.051*	
C11	0.55428 (15)	0.6166 (3)	0.14712 (7)	0.0517 (5)	
H11A	0.5937	0.5141	0.1336	0.078*	
H11B	0.6039	0.6927	0.1664	0.078*	
H11C	0.5231	0.6837	0.1185	0.078*	
N1	0.37789 (11)	0.38698 (19)	0.11129 (5)	0.0406 (4)	
H1	0.4407 (8)	0.369 (2)	0.0965 (6)	0.046 (5)*	
O1	0.19506 (10)	0.3884 (2)	0.09562 (5)	0.0656 (4)	
O2	0.06813 (10)	0.2998 (3)	0.02376 (5)	0.0746 (5)	
H2A	0.1092 (19)	0.340 (3)	0.0519 (7)	0.098 (8)*	
O3	0.08638 (11)	0.2036 (2)	−0.05732 (5)	0.0700 (4)	
Cl1	0.38181 (4)	0.61481 (7)	0.334722 (17)	0.0585 (2)	

### Atomic displacement parameters (Å^2^)

**Table:**

	*U*^11^	*U*^22^	*U*^33^	*U*^12^	*U*^13^	*U*^23^
C1	0.0340 (9)	0.0542 (10)	0.0401 (9)	−0.0017 (8)	−0.0029 (7)	−0.0048 (8)
C2	0.0339 (9)	0.0681 (12)	0.0436 (10)	−0.0021 (8)	0.0016 (7)	−0.0083 (9)
C3	0.0436 (9)	0.0720 (12)	0.0359 (9)	−0.0057 (9)	0.0018 (8)	−0.0065 (9)
C4	0.0438 (10)	0.0691 (12)	0.0358 (10)	−0.0091 (9)	−0.0067 (8)	0.0037 (9)
C5	0.0355 (8)	0.0380 (8)	0.0370 (9)	0.0011 (7)	−0.0049 (7)	−0.0031 (7)
C6	0.0355 (8)	0.0360 (8)	0.0434 (9)	−0.0009 (7)	−0.0029 (7)	−0.0012 (7)
C7	0.0441 (10)	0.0389 (9)	0.0463 (10)	−0.0041 (8)	−0.0090 (8)	−0.0061 (7)
C8	0.0509 (10)	0.0366 (8)	0.0356 (9)	0.0025 (8)	−0.0047 (7)	−0.0026 (7)
C9	0.0473 (10)	0.0460 (9)	0.0415 (9)	−0.0040 (8)	0.0030 (7)	0.0015 (8)
C10	0.0410 (9)	0.0452 (9)	0.0425 (9)	−0.0094 (8)	−0.0028 (7)	−0.0027 (7)
C11	0.0440 (10)	0.0592 (11)	0.0519 (11)	−0.0133 (9)	0.0002 (8)	−0.0048 (9)
N1	0.0321 (8)	0.0517 (9)	0.0380 (8)	−0.0033 (6)	−0.0008 (6)	−0.0070 (6)
O1	0.0350 (7)	0.1178 (13)	0.0441 (7)	0.0058 (7)	−0.0029 (5)	−0.0196 (7)
O2	0.0374 (7)	0.1425 (15)	0.0439 (8)	−0.0093 (8)	−0.0041 (6)	−0.0140 (9)
O3	0.0527 (8)	0.1129 (12)	0.0444 (7)	−0.0126 (8)	−0.0140 (6)	−0.0092 (8)
Cl1	0.0767 (4)	0.0599 (3)	0.0388 (3)	−0.0056 (2)	−0.0028 (2)	−0.00955 (19)

### Geometric parameters (Å, °)

**Table:**

C1—O1	1.238 (2)	C6—C11	1.510 (2)
C1—O1	1.238 (2)	C7—C8	1.382 (2)
C1—N1	1.335 (2)	C7—H7	0.9300
C1—C2	1.482 (2)	C8—C9	1.375 (2)
C2—C3	1.333 (2)	C8—Cl1	1.7478 (16)
C2—H2	0.9300	C9—C10	1.386 (2)
C3—C4	1.479 (3)	C9—H9	0.9300
C3—H3	0.9300	C10—H10	0.9300
C4—O3	1.213 (2)	C11—H11A	0.9600
C4—O2	1.308 (2)	C11—H11B	0.9600
C5—C10	1.389 (2)	C11—H11C	0.9600
C5—C6	1.400 (2)	N1—H1	0.861 (5)
C5—N1	1.429 (2)	O1—O1	0.000 (5)
C6—C7	1.394 (2)	O2—H2A	0.920 (5)
			
O1—C1—O1	0.00 (12)	C8—C7—H7	119.6
O1—C1—N1	122.22 (16)	C6—C7—H7	119.6
O1—C1—N1	122.22 (16)	C9—C8—C7	121.03 (15)
O1—C1—C2	123.26 (15)	C9—C8—Cl1	119.60 (13)
O1—C1—C2	123.26 (15)	C7—C8—Cl1	119.37 (13)
N1—C1—C2	114.50 (14)	C8—C9—C10	119.05 (16)
C3—C2—C1	129.17 (16)	C8—C9—H9	120.5
C3—C2—H2	115.4	C10—C9—H9	120.5
C1—C2—H2	115.4	C9—C10—C5	120.47 (15)
C2—C3—C4	131.75 (17)	C9—C10—H10	119.8
C2—C3—H3	114.1	C5—C10—H10	119.8
C4—C3—H3	114.1	C6—C11—H11A	109.5
O3—C4—O2	121.08 (16)	C6—C11—H11B	109.5
O3—C4—C3	118.71 (17)	H11A—C11—H11B	109.5
O2—C4—C3	120.21 (15)	C6—C11—H11C	109.5
C10—C5—C6	120.67 (15)	H11A—C11—H11C	109.5
C10—C5—N1	120.95 (14)	H11B—C11—H11C	109.5
C6—C5—N1	118.36 (14)	C1—N1—C5	126.65 (14)
C7—C6—C5	117.92 (15)	C1—N1—H1	114.9 (12)
C7—C6—C11	120.05 (15)	C5—N1—H1	118.4 (12)
C5—C6—C11	122.03 (15)	O1—O1—C1	0(10)
C8—C7—C6	120.85 (15)	C4—O2—H2A	113.1 (17)
			
O1—C1—C2—C3	−3.2 (3)	C6—C7—C8—Cl1	−179.45 (13)
O1—C1—C2—C3	−3.2 (3)	C7—C8—C9—C10	0.9 (3)
N1—C1—C2—C3	178.4 (2)	Cl1—C8—C9—C10	−179.48 (13)
C1—C2—C3—C4	−2.4 (4)	C8—C9—C10—C5	−1.1 (3)
C2—C3—C4—O3	−176.3 (2)	C6—C5—C10—C9	0.2 (3)
C2—C3—C4—O2	2.7 (4)	N1—C5—C10—C9	178.60 (15)
C10—C5—C6—C7	0.8 (2)	O1—C1—N1—C5	1.3 (3)
N1—C5—C6—C7	−177.59 (14)	O1—C1—N1—C5	1.3 (3)
C10—C5—C6—C11	−178.68 (16)	C2—C1—N1—C5	179.73 (16)
N1—C5—C6—C11	2.9 (2)	C10—C5—N1—C1	34.9 (3)
C5—C6—C7—C8	−1.0 (2)	C6—C5—N1—C1	−146.67 (17)
C11—C6—C7—C8	178.52 (16)	N1—C1—O1—O1	0.00 (10)
C6—C7—C8—C9	0.1 (3)	C2—C1—O1—O1	0.00 (4)

### Hydrogen-bond geometry (Å, °)

**Table:**

*D*—H···*A*	*D*—H	H···*A*	*D*···*A*	*D*—H···*A*
N1—H1···O3^i^	0.86 (1)	2.10 (1)	2.9556 (19)	174.(2)
O2—H2A···O1	0.92 (1)	1.57 (1)	2.4797 (17)	171 (3)

Symmetry codes: (i) *x*+1/2, −*y*+1/2, −*z*.
